# HIV-1 Sub-subtype A6 Remains Susceptible to Second-Generation Integrase Inhibitors With Limited Emergence of Resistance in Vitro

**DOI:** 10.1093/ofid/ofag230

**Published:** 2026-04-21

**Authors:** Francesco Saladini, Alessia Giannini, Federica Giammarino, Anastasiia Antonova, Ekaterina Ozhmegova, Rolf Kaiser, Filippo Dragoni, Adele Boccuto, Ilaria Vicenti, Anders Sönnerborg, Marina Bobkova, Maurizio Zazzi

**Affiliations:** Department of Medical Biotechnologies, University of Siena, Siena, Italy; Department of Medical Biotechnologies, University of Siena, Siena, Italy; Department of Medical Biotechnologies, University of Siena, Siena, Italy; Federal State Budget Institution “National Research Center for Epidemiology and Microbiology Named After the Honorary Academician N.F. Gamaleya,” Ministry of Health of the Russian Federation, Moscow, Russia; Federal State Budget Institution “National Research Center for Epidemiology and Microbiology Named After the Honorary Academician N.F. Gamaleya,” Ministry of Health of the Russian Federation, Moscow, Russia; Institute of Virology, University of Cologne, Faculty of Medicine and University Hospital of Cologne, Cologne, Germany; Department of Medical Biotechnologies, University of Siena, Siena, Italy; Department of Medical Biotechnologies, University of Siena, Siena, Italy; Department of Medical Biotechnologies, University of Siena, Siena, Italy; Department of Clinical Microbiology, Karolinska University Hospital, Stockholm, Sweden; Department of Medicine Huddinge, Division of Infectious Diseases, Karolinska Institutet, Stockholm, Sweden; I.Mechnikov Research Institute for Vaccines and Sera, Moscow, Russia; Department of Medical Biotechnologies, University of Siena, Siena, Italy

**Keywords:** bictegravir, cabotegravir, dolutegravir, HIV-1, INSTI resistance, sub-subtype A6

## Abstract

**Background:**

HIV-1 sub-subtype A6 is predominant in Eastern Europe and was associated with increased risk of treatment failure with the long-acting cabotegravir plus rilpivirine regimen. In this study, we aimed to evaluate the in vitro susceptibility and the genetic barrier to resistance to INSTI in recombinant viruses harboring clinically derived A6 integrase coding regions.

**Methods:**

We generated 23 NL4-3 strain-based recombinant viruses harboring clinically derived integrase coding region. We measured their susceptibility to second-generation INSTIs dolutegravir, bictegravir, and cabotegravir in a TZM-bl cell-based phenotypic assay. The genetic barrier to resistance was evaluated by exposing MT-2 cell cultures infected with 4 A6 integrase recombinant viruses, as well as the NL4-3 and HXB2 subtype B reference strains.

**Results:**

All 23 recombinant viruses generated with clinically derived A6 integrase displayed full susceptibility to dolutegravir, bictegravir, and cabotegravir, showing median (interquartile range) fold-change values of 1.2 (0.9–1.5), 1.1 (0.7–1.5), and 0.9 (0.6–1.1), respectively. Of 4 A6 viruses assessed for their genetic barrier to resistance in vitro, only 1 showed emerging integrase mutations E138K or Q148R at subinhibitory concentrations of dolutegravir or cabotegravir, respectively.

**Conclusions:**

These data suggest that sub-subtype A6 integrase has full susceptibility and largely maintains a high genetic barrier to resistance to second-generation integrase strand transfer inhibitors.

## Teaser

HIV-1 sub-subtype A6 was tested for susceptibility and resistance selection to second-generation INSTIs. Twenty-three A6 integrase recombinant viruses showed full susceptibility, with resistance emerging only in one isolate, indicating A6 integrase maintains a high genetic barrier to INSTI resistance.

The genetic diversity of the HIV type 1 (HIV-1) has significant implications for viral transmission dynamics, disease progression, and antiretroviral therapy (ART) outcomes. HIV-1 group M, responsible for the global pandemic, comprises several subtypes (A–D, F–H, J–L), sub-subtypes, and circulating recombinant forms (CRFs), each with distinct geographic distributions and molecular characteristics [1]. Among the multiple subtype A lineages (A1–A8), sub-subtype A6 was first detected in the former Soviet Union in the late 1990s, likely introduced through limited founder events, followed by rapid expansion first among people who inject drugs [2] and later within the heterosexual population [3]. Since then, A6 has become the most prevalent HIV-1 variant in Russia and several neighboring countries, and displacement of the Ukrainian population due to the ongoing conflict with Russia has further increased A6 spread [4].

HIV-1 sub-subtype A6 has recently raised concerns regarding its potential impact on the long-acting injectable regimen including the non-nucleoside reverse transcriptase inhibitor rilpivirine and the integrase strand transfer inhibitor (INSTI) cabotegravir. Indeed, data from clinical trials have shown that the presence of at least 2 baseline factors among sub-subtype A6, proviral rilpivirine resistance-associated mutations and higher body mass index, was significantly associated with an increased risk of virological failure [5]. Sub-subtype A6 is characterized by the presence of the natural integrase polymorphism L74I as consensus amino acid [6]. In vitro analyses on site-directed mutants demonstrated that L74I does not decrease the susceptibility to cabotegravir but suggested that it may facilitate cabotegravir resistance by increasing the replication capacity of the viral strains harboring INSTI resistance mutations, but not that of wild-type viruses [7, 8]. To further characterize the possible role of the A6 sub-subtype in response to the second-generation INSTI cabotegravir, dolutegravir, and bictegravir, we evaluated the in vitro susceptibility and emergent resistance to INSTI in recombinant viruses harboring clinically derived A6 integrase coding regions.

## METHODS

### Cell Lines and Antiretroviral Drugs

Lenti-X 293 T cells (Takara Bio, Japan) and TZM-bl cells were cultured in high-glucose DMEM with L-glutamine, supplemented with 10% FBS, 100 U/mL penicillin, and 100 µg/mL streptomycin. The MT-2 HTLV-1 transformed lymphoblastoid cell line was cultured in RPMI supplemented with 2 mM L-glutamine, 10% FBS, 100 U/mL penicillin, and 100 µg/mL streptomycin. TZM-bl and MT-2 cell lines, together with the INSTIs dolutegravir, bictegravir, and cabotegravir, were obtained from the Centre for AIDS Reagent of the National Institute for Biological Standards and Control. All cell culture media and reagents were obtained from EuroClone (Italy).

### Generation of HIV-1 Recombinant Viruses

The generation of recombinant viruses harboring clinically derived integrase coding region has been previously described [9]. Briefly, viral RNA was collected from people with HIV-1 never exposed to INSTI and extracted through the EZ1 automatic system and the DSP Virus Kit (Qiagen) according to the manufacturer's instructions. Viruses were previously classified as sub-subtype A6 through phylogenetic analysis on HIV-1 pol sequences obtained for routine genotypic testing for monitoring of drug resistance. Sample sequences were aligned with representative sequences of each subtype including A6 retrieved from the HIV Database of the Los Alamos National Laboratory (https://www.hiv.lanl.gov/). Phylogenetic tree was built with the maximum likelihood method using MEGA 7 software. Sequences were attributed to sub-subtype A6 according to their clustering within A6 reference sequences included in the data set. The use of residual plasma for research purposes was based on the patient's informed consent as approved by the local Ethics Committee of the Siena University Hospital. Reverse transcription from 20 µL of extracted RNA was performed using the Improm-II Reverse Transcriptase (Promega), while amplification of the whole integrase coding region (nucleotide coordinates on the HXB2 reference genome 4009–5219) was performed using the Q5 High-Fidelity DNA Polymerase (NEB) according to the manufacturer's instructions in a 2-step polymerase chain reaction (PCR) approach. Triplicate nested PCR was co-transfected with the correspondingly deleted and linearized pNL4-3ΔIN in Lenti-X 293 T cells using calcium-phosphate. Supernatants harboring recombinant viruses were harvested 48 hours post-transfection and expanded in MT-2 cells to increase viral titers. In presence of large cellular syncytia, supernatants were harvested and stored at −80°C.

### Determination of Phenotypic Susceptibility to INSTI

Susceptibility to dolutegravir, bictegravir, and cabotegravir was evaluated by quantifying luciferase activity after TZM-bl infection with recombinant viruses in the presence of serial dilutions of each drug as previously described [9]. To determine the IC_50_ of each recombinant virus, TZM-bl cells were seeded in a 96-well plate at concentration of 10 000 cells per well and infected with 300 TCID_50_ of virus in the presence of 5-fold serial dilutions of each INSTI. After 48 hours, cells were lysed, adding 40 µL/well of Glo-Lysis Buffer (Promega), and the lysate was transferred to a luminescence plate. Forty microliters of Bright-Glo Luciferase Reagent (Promega) was added to each well, and luminescence was measured in the Glomax Discover platform (Promega). Relative Luminescence Units (RLU) detected in each well were elaborated with GraphPad Prism, version 9.0 (GraphPad Software), to calculate the IC_50_ values of recombinant and wild-type viruses. Fold-change (FC) values were calculated as the ratio between the IC_50_ of each A6 integrase recombinant virus and the IC_50_ of the NL4-3 subtype B wild-type virus. Given the excellent correlation between this system and the de facto standard Phenosense assay [9], FC values were interpreted based on the clinical and biological cutoff values established in the Phenosense methodology. Briefly, lower and upper clinical cutoffs (4 and 13, respectively) have been defined for DTG based on clinical trials in pretreated subjects harboring drug-resistant virus [10], while BIC and CAB have not been used in such populations; thus only a biological cutoff of 2.5 has been defined as the mean fold-change plus 2 standard deviations when analyzing drug unexposed viruses [11].

### In Vitro Resistance Selection Experiments

Four A6 integrase recombinant viruses derived from distinct individuals, as well as the NL4-3 and HXB2 wild-type reference strains, were used to infect MT-2 cells in presence of either dolutegravir, cabotegravir, or bictegravir at the respective IC_90_ value as calculated with the NL4-3 strain. At the time of viral breakthrough, defined as the day of the appearance of cellular syncytia, the supernatant was clarified by centrifugation at 300*g* for 10 minutes and used for both Sanger sequencing and the infection of a new MT-2 culture in the presence of a 2-fold higher drug concentration. Cell cultures were interrupted 74 days after experiment initiation. Sequencing was performed on both the integrase coding region and the 3′ polypurine tract (3′-PPT).

## RESULTS

**Figure 1.**
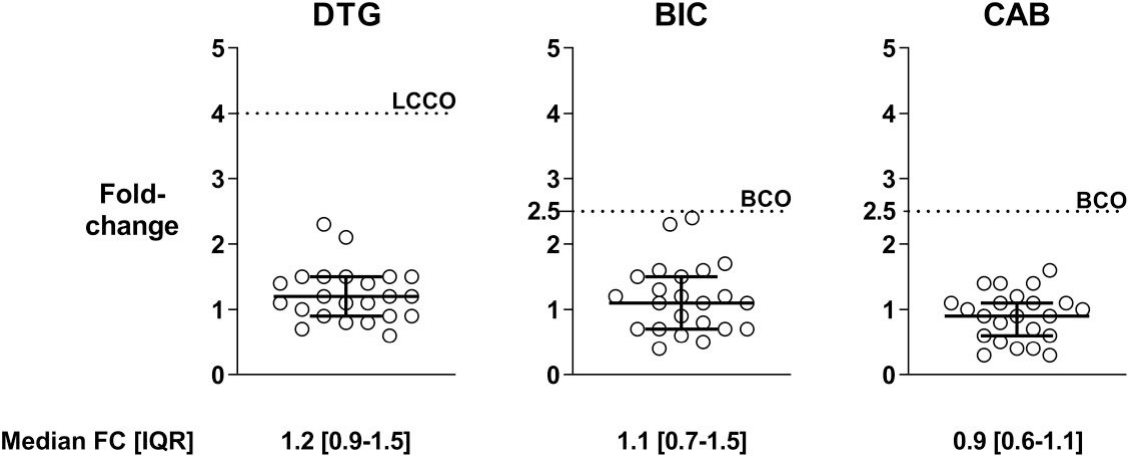
Fold-change values of recombinant viruses harboring clinically derived sub-subtype A6 integrase calculated with dolutegravir, bictegravir, and cabotegravir. BCO indicates the biological fold-change cutoff value, while LCCO indicates the lower clinical fold-change cutoff value as determined through the Phenosense Integrase Assay (Monogram Biosciences). Abbreviations: BCO, biological fold-change cutoff value; BIC, bictegravir; CAB, cabotegravir; DTG, dolutegravir; FC, fold-change; IQR, interquartile range; LCCO, lower clinical fold-change cutoff.

We successfully generated 23 A6 integrase recombinant viruses from residual plasma samples that underwent routine drug resistance testing. None of the viruses harbored major INSTI resistance-associated mutations as defined by the Stanford HIV Drug Resistance Database (https://hivdb.stanford.edu/), while 22/23 (96%) sequences had the L74I variant, which is the consensus amino acid in sub-subtype A6. Median fold-change values (interquartile range [IQR]) determined by phenotypic testing in TZM-bl cells were 1.2 (0.9–1.5), 1.1 (0.7–1.5), and 0.9 (0.6–1.1) for dolutegravir, bictegravir, and cabotegravir, respectively. According to the biological or clinical fold-change cutoffs established with the Phenosense Integrase assay, all the A6 recombinant viruses had full susceptibility to dolutegravir, bictegravir, and cabotegravir (Figure 1).

**Figure 2.**
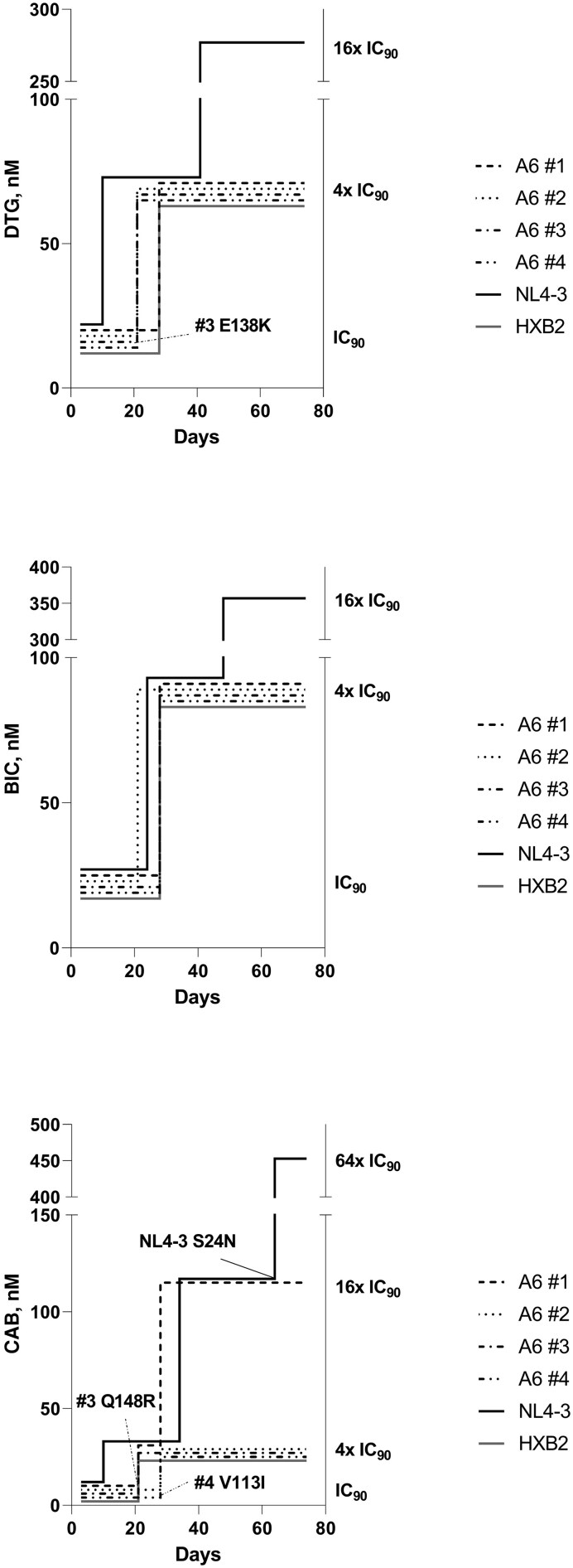
In vitro resistance selection experiments with recombinant viruses harboring clinically derived sub-subtype A6 integrase (numbers 1 to 4), as well as with the NL4-3 and HXB2 reference subtype B viruses exposed to increasing concentrations of dolutegravir, bictegravir, and cabotegravir. Experiments started using the 90% inhibitory concentration for each drug as calculated with the NL4-3 virus. Emerging mutations within the integrase coding region at viral breakthrough are indicated in the graphs. Abbreviations: BIC, bictegravir; CAB, cabotegravir; DTG, dolutegravir; IC_90_, 90% inhibitory concentration.

In vitro resistance selection experiments were performed using 4 A6 integrase recombinant viruses carrying either leucine (virus 1) or isoleucine (viruses 2, 3, 4) at integrase position 74 (Figure 2). All A6 viruses showed comparable time to viral breakthrough at dolutegravir, bictegravir, and cabotegravir IC_90_ (days 21–28 postinfection). The NL4-3 virus showed an earlier viral breakthrough (day 10 postinfection) without any emerging mutation when exposed to dolutegravir and cabotegravir. The NL4-3 virus was also able to replicate at dolutegravir, bictegravir, and cabotegravir 4× IC_90_ in the absence of emerging mutations in both integrase and 3′-PPT, while escaped cabotegravir 16× IC_90_ with the emerging S24N integrase mutation. Among the A6 recombinant viruses, virus 3 showed emerging integrase mutations E138K or Q148R when exposed to dolutegravir IC_90_ or cabotegravir IC_90_, respectively, while emerging integrase mutation V113I was detected in virus 4 when exposed to cabotegravir IC_90_. No other emerging mutations within the integrase and 3′-PPT regions were identified for any other recombinant viruses or HXB2 virus when exposed to dolutegravir, bictegravir, and cabotegravir IC_90_. While E138K and Q148R are known resistance-associated mutations affecting INSTI susceptibility, an analysis performed on 5381 HIV-1 group M integrase sequences retrieved from the HIV Sequence Database of the Los Alamos National Laboratory (LANL; https://www.hiv.lanl.gov/) revealed that asparagine at position 24 is a natural polymorphism with a prevalence of 7.5%, while valine and isoleucine at position 113 occur with a prevalence of 19.0% and 80.4%, respectively. All A6 integrase recombinant viruses and NL4-3 virus carried valine at position 113, while only the HXB2 virus carried isoleucine at this site.

## DISCUSSION

Following the suggestion of a role of HIV-1 sub-subtype A6 in the response to long-acting cabotegravir and rilpivirine, initial studies explored the impact of the integrase natural polymorphism L74I on replication capacity and development of resistance to cabotegravir [7, 8]. Our study provides additional information on natural susceptibility and the genetic barrier to resistance of sub-subtype A6 against second-generation INSTI. Unlike previous studies using single molecular clones, the generation of a panel of recombinant viruses harboring clinically derived integrase coding regions in our study addressed the possible contribution of genetic variability within the A6 sub-subtype. Phenotypic assessment of a panel of 23 A6 strains showed that A6 viruses have the same susceptibility to second-generation INSTI as that of the NL4-3 wild-type virus, with fold-change values below the biological or clinical fold-change cutoff. While divergent individual isolates may occasionally occur for any HIV-1 subtype, these data support that natural polymorphisms in A6 integrase do not impact INSTI susceptibility.

In vitro selection experiments were performed using 3 randomly selected recombinant A6 viruses harboring the consensus integrase L74I polymorphism and the only A6 recombinant virus with leucine at position 74. The experiments showed that all viruses replicated at subinhibitory drug concentration corresponding to the IC_90_ value but failed to replicate at higher doses, except for the NL4-3 virus with all drugs and the A6 integrase recombinant virus 1 with cabotegravir. The high-fit, pure-subtype, and laboratory-adapted NL4-3 virus may have had higher replication capacity in our experimental conditions. Notably, 1 of the 4 A6 integrase recombinants evaluated in resistance selection experiments (virus 3) showed emerging mutations at sub-inhibitory doses of cabotegravir (Q148R) and dolutegravir (E138K), resembling in vivo data. Indeed, other studies have shown that Q148R typically emerged both during in vitro drug pressure and at cabotegravir treatment failure [12], while E138K usually emerged together with other major mutations at codon 148 during exposure to dolutegravir [13]. Both mutations may have had a limited impact on drug susceptibility and/or suboptimal fitness in our experimental conditions, possibly explaining the lack of replication at 4× IC_90_ cabotegravir or dolutegravir. Cabotegravir has been suggested to have a lower genetic barrier to resistance with respect to other second-generation INSTIs [14]. However, previous in vitro resistance selection experiments showed comparable rates of viral breakthrough with cabotegravir and dolutegravir [14]. Thus, it remains unclear if and how the long-acting formulation plays a role in lowering the genetic barrier to cabotegravir resistance in vivo. This has important implications in the context of the expanding use of the injectable combination of cabotegravir and rilpivirine, including in viremic individuals with low adherence to oral ART [15], as well as with cabotegravir pre-exposure prophylaxis [16].

The early selection of the E138K mutation in A6 recombinant virus 3 might suggest a reduced genetic barrier in the context of A6 integrase, at least at a subinhibitory dolutegravir concentration. However, such phenotype may be associated with the specific virus 3 integrase genotype, and it is currently not possible to derive any further conclusions based on the low number of isolates tested here. However, clinical data from resource-limited countries demonstrate that resistance to dolutegravir may occur more frequently than previously shown, particularly with likely reduced NRTI backbone activity or inadequate adherence, with a possible increased risk with non-B subtypes [17]. Notably, we did not detect any emergent resistance to bictegravir, making this apparent divergence worth further analysis.

A major limitation of this study, similar to previous in vitro studies, is the generation of chimeric viruses harboring clinical A6 integrase in the genetic background of the NL4-3 subtype B genome. While all the recombinants replicated well under our experimental conditions, this strategy may have had an unpredictable impact on the interaction between integrase and other viral proteins, resulting in altered replication capacity. Further studies using fully A6 clinical isolates, together with strains from subtype B and other non-B subtypes, are needed to formally investigate the impact of the whole genetic background on susceptibility to INSTI.

The experiments were conducted for ∼11 weeks, which might have limited the emergence of resistance in vitro. Indeed, previous in vitro experiments have shown that the selection of resistance to second-generation INSTIs in vitro may require longer exposure [14, 18]. However, in vitro experiments have shown that cabotegravir selects mutations, resulting in decreased susceptibility after 8 weeks of drug exposure, indicating that a limited duration of experiments may be sufficient for the detection of viral adaptation to drug pressure [14, 19].

In conclusion, our findings indicate that HIV-1 sub-subtype A6 integrase retains full susceptibility to second-generation INSTIs and suggest that the cabotegravir genetic barrier, as assessed in formal in vitro selection experiments, may not explain the high rate of emergent resistance at treatment failure in vivo, although specific viral genetic backgrounds may facilitate certain resistance pathways. This points to a specific role of the long-acting formulation through currently unexplained mechanisms. Also, continued surveillance and resistance monitoring during INSTI-based regimens are warranted, particularly with the changing pattern of HIV-1 subtype circulation and the evolution of treatment strategies.
